# Supplementary material for: Joint Selenium–Iodine Supply and Arbuscular Mycorrhizal Fungi Inoculation Affect Yield and Quality of Chickpea Seeds and Residual Biomass
Source: Plants (Basel). 2020 Jun 27;9(7):804. doi: 10.3390/plants9070804 (PMC7412542; doi:10.3390/plants9070804)
Supplement: Supplementary file 1 [file plants-09-00804-s001.pdf]

Supplemental Figures

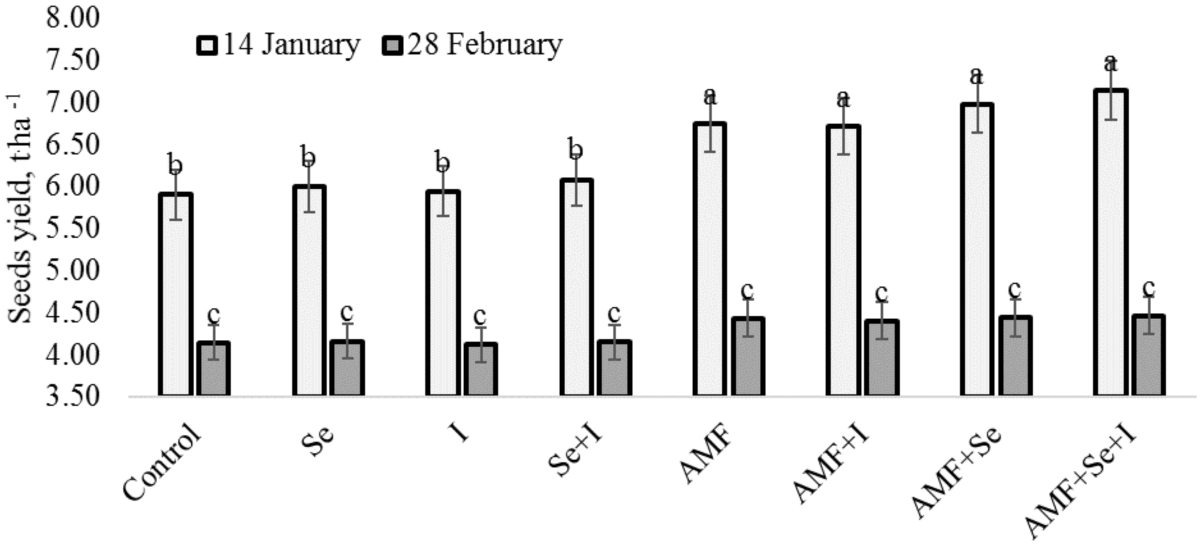

Figure S1. Interaction between planting time and AMF biofortification on the seed yield. Values followed by different letters are significantly different according to Duncan's test at $p \leq 0.05$.

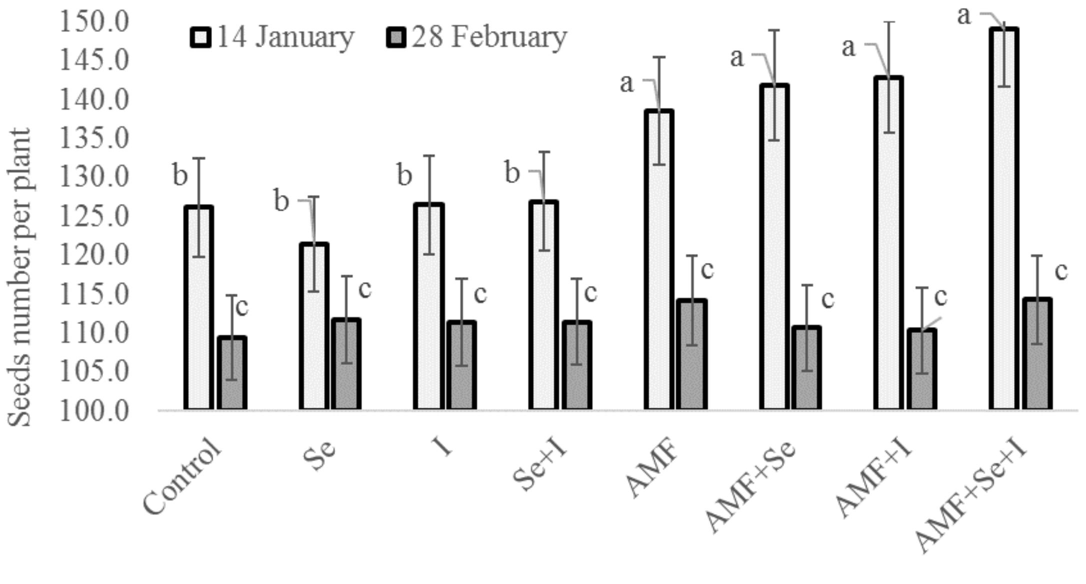

Figure S2. Interaction between planting time and AMF biofortification on the seed number per plant. Values followed by different letters are significantly different according to Duncan's test at $p \leq 0.05$.

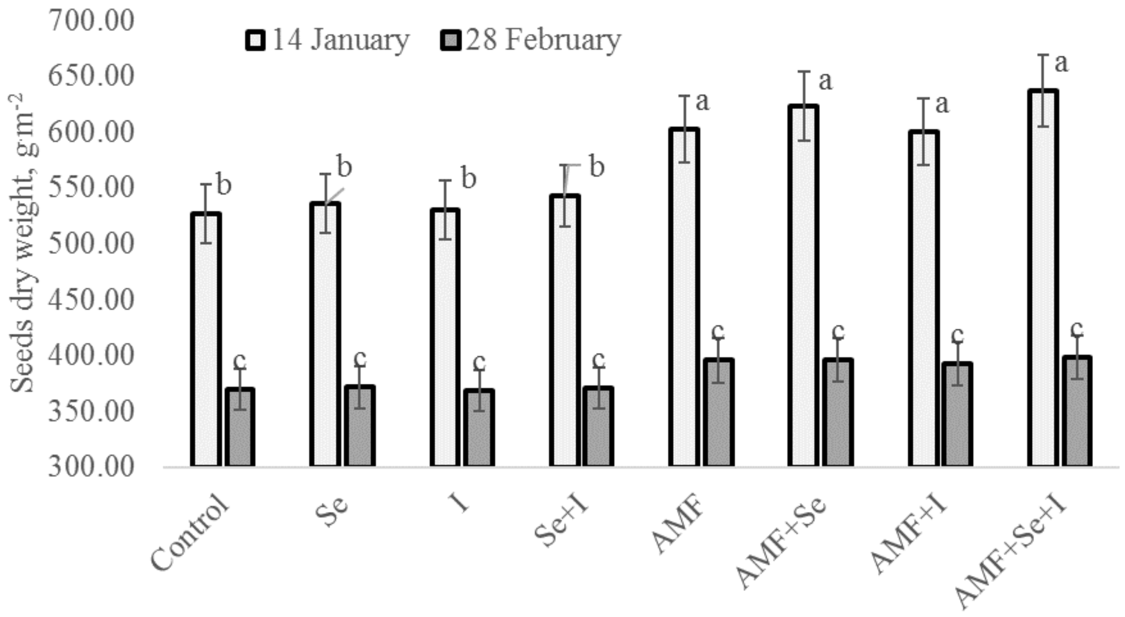

Figure S3. Interaction between planting time and AMF biofortification on seed dry weight. Values followed by different letters are significantly different according to Duncan's test at $p \leq 0.05$.

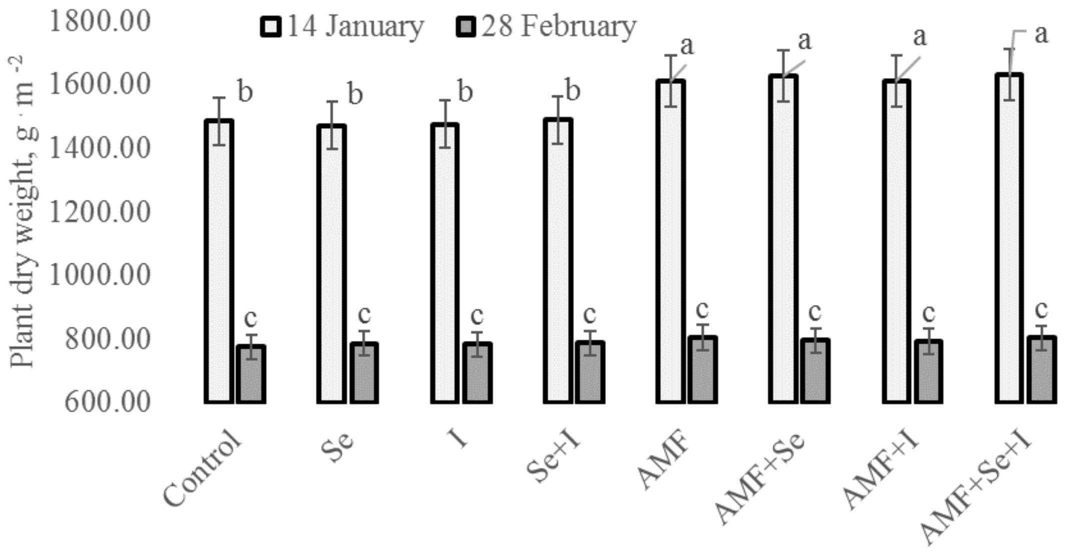

Figure S4. Interaction between planting time and AMF biofortification on plant dry weight. Values followed by different letters are significantly different according to Duncan's test at $p \leq 0.05$.
